# Supplementary material for: Assessment of heavy metals in soil and water from Bahi district, Tanzania
Source: PLoS One. 2025 Jun 11;20(6):e0325487. doi: 10.1371/journal.pone.0325487 (PMC12157122; doi:10.1371/journal.pone.0325487)
Supplement: S1 Table — (PDF) [file pone.0325487.s001.pdf]

Description of ALSMatriX-GpDN Sampling tool

A : Area code (Initial of a District):

Example: **B** for Bahi district

L: Location code (initial of village/street/ward):

Example: **I** for Ilindi village

S (Sample Type):

S = Soil, **W** = Water, **C** = Cereal, M= Maize etc.

Matri: Description of sampling point (matrix of the soil or water) :

1. Soil: 0 = Uncultivated, 1 = Cultivated
2. Water: 0 = Pond, 1 = Stream, 2 = Swamp, 3 = Well, 4 = river, 5= lake, etc

X (Site/ Field ID):

Numerical identifier for field (e.g., 01, 02, 03) are sequential together with a name. e.g. the first field where sample was collected was from Mr. XYZ, hence X is assigned 01 with which several samples can be collected from different points, Gp.

Gp (Geographical point):

Number indicating each sample collected at the same field for the purpose of duplicate/triplicate collection (e.g., 1, 2, 3). GPS coordinate must be recorded where applicable.

D (Depth Code):

1. Soil: 0 = Unspecified, 1 = 0-5 cm, 2 = 6-15 cm, 3 = 16-30 cm
2. Water: 0 = Surface, 1 = Shallow, 3 = Deeper, can be improved

N (Sequential Sample Number):

Indicates the sample number collected in sequence (e.g., 03 for third sample)

S1A Table. ***ALSMtriX-GpDN*** **tool for soil samples**

Sample Type: ………………………………………………….   Date of Sample collection: ……………………………

Region: ……………………………………   District …………………………………….. Ward/Street/Village ……………………………..

Project/Title: ……

Sample Code	*REPORTING CODE*	*Area District (A)*	*Location Site/village/street (L)*	*Sample Type (S)*	*Description of sample matrix (Matri)*	*Site/ Field ID (X)*	*Geographical Point Number (Gp)*	*Depth (D)*	*Sample S/N (N)*	*GPS Coordinates (Latitude, Longitudes)*	*Additional Notes*
BIS001-1101	*BIS01*	*Bahi*	*Ilindi*	*Soil (S)*	*0*	*01*	*1*	*1*	*01*	*-6.1245, 35.5123*	*Collected from farm*
BIS001-2102	*BIS02*	*Bahi*	*Ilindi*	*S*	*0*	*01*	*2*	*1*	*02*	*-6.1245, 35.5123*	*Collected from farm*
BIS001-3103	*BIS03*	*Bahi*	*Ilindi*	*S*	*0*	*01*	*3*	*1*	*03*	*-6.1245, 35.5123*	*Collected from farm*

Name of Sample collector/researcher ……………………………… Signature ……………………………….. Date: …………………………….

S1B Table. ALSMtriX-GpDN tool for soil samples

Sample Type: …………………………………………………. Date of Sample collection: ……………………………

Region: ……………………………………… District …………………………………….. Ward/Street/Village ……………………………..

Project/Title: ……

Sample Code	*REPORTING CODE*	*Area District (A)*	*Location Site/village/street (L)*	*Sample Type (S)*	*Description of sample matrix (Matri)*	*Site/ Field ID (X)*	*Geographical Point Number (Gp)*	*Depth (D)*	*Sample S/N (N)*	*GPS Coordinates (Latitude, Longitudes)*	*Additional Notes*
MMW102-2205	*MMW01*	*Manyoni*	*Mbugani*	*Water (W)*	*1*	*02*	*1*	*1*	*01*	*-5.7823, 34.9054*	*Near water source*

Name of Sample collector/researcher ……………………………… Signature ……………………………….. Date: …………………………….

S1C Table. Optimal Conditions for AAS Analysis of Mn, Zn, and Cu

Element	Wavelength (nm)	Lamp Current (mA)	Slit (nm)	Flow (L/min)	Burner Height (mm)
Mn	279.5	5	0.2	2.5	7
Zn	213.9	8	0.7	2.5	7.5
Cu	324.7	6	0.7	2.5	6.5

S1D Table. Harmonized Optimal Digestion Condition

Parameter	Optimal Value
HNO_3 (mL)	7–8
HCl (mL)	4–5
Total Volume (mL)	12
Temperature Range	110–170°C
Pressure	45–75 psi
Time	40 minutes
Observation	Clear and pale solution.

S1E Table. LoD, Quality Control and Certified Reference standard used during ED-XRF measurements

Element	Limit of Detection (LoD) (mg/kg)	Accuracy on Internal Quality Control (%)	Recovery on SRM 2711A Certified Reference (%)
Pb	2.0	95.0 ± 3.5	92.0 ± 2.6
Cd	1.5	94.0 ± 4.0	91.0 ± 3.5
Mn	5.0	96.5 ± 2.0	97.0 ± 1.8
Zr	10.0	93.0 ± 3.5	89.5 ± 2.5
Cu	3.0	95.0 ± 2.5	94.0 ± 2.2
As	2.5	92.5 ± 3.5	90.0 ± 2.5
Zn	4.0	96.0 ± 2.0	95.5 ± 1.6
Sr	5.0	93.0 ± 4.0	92.0 ± 2.5
Cs	1.8	94.5 ± 3.0	89.0 ± 3.0

S1F Table. Geographical coordinated corresponding to the mean concentrations (mg/kg) of heavy metals is soil samples

Sample	Longitude	Latitude	Pb	Cd	Mn	Zr	Cu	As	Zn	Sr	Cs
BNS2	35.2631667	-6.0031667	21	62	448	198	40	2	33	146	123
BNS3	35.2593333	-6.0041833	47	46	576	228	55	2	54	153	135
BNS4	35.2593333	-6.0041833	34	46	540	220	47	7	51	158	139
BCS5	35.3047667	-5.9418	25	65	267	71	65	3	19	78	135
BCS6	35.3047667	-5.9418	22	67	234	55	53	5	18	81	152
BCS7	35.304995	-5.94551	19	65	313	169	57	5	28	72	158
BTS8	35.279852	-6.014628	39	38	882	161	57	9	101	135	120
BTS9	35.290021	-6.001490,	38	37	883	160	56	8	102	136	122
BTS92	35.2974	-5.9417667	40	39	881	162	58	7	100	134	121
BMS10	35.3266833	-6.0484333	25	705	335	204	46	7	31	95	154
BMS11	35.3266833	-6.0484333	21	706	219	275	57	6	22	80	134
BMS12	35.3266833	-6.0484333	20	704	366	278	45	3	33	90	136
BSS13	35.3137833	-5.9500833	31	1156	409	303	49	5	45	158	126
BSS14	35.3056833	-6.0286333	30	1157	410	304	48	4	44	156	127
BSS15	35.313845	-5.950113	32	1158	411	302	47	6	46	157	128

Soil samples from Bahi Sokoni (BS), Bahi Matajila (BT), Bahi Chang'ombe (BC), Bahi Nagulolu (BN), Bahi Makulu (BM)

S1G Table. Geographical coordinated corresponding to the mean concentrations (mg/L) of Mn and Zn water samples

Code	Station	Longitude	Latitude	Mn	zn
**W-01**	RB	35.30508333	-5.94245	0.65	< 0.01
**W-03**	RB	35.307554	-5.939674	0.5	< 0.01
**W-05**	BS	35.25933333	-6.004183333	0.21	< 0.01
**W-07**	BS	35.310592	-5.950021	0.38	0.1
**W-09**	RM	35.32668333	-6.048433333	0.27	0.12
**W-13**	BSo	35.31378333	-5.950083333	0.25	0.18
**W-14**	BSo	35.312999	-5.951007	0.51	0.13
	Mean			0.33	0.09
	SD			0.2	0.08
	Sum			2.34	0.64
	Min			0.05	0.01
	Max			0.64	0.19

Water samples; River Bubu (W-01), Bahi Swamp (W-03), Bahi town (W-05), Chali town (W-07); Bahi Makulu (W-09), Bahi Sokoni (W-013) and Bahi sokoni (W-014) Value reported as mean ± SD; n = 3. BDL = Below detectable limit.
