# Supplementary material for: Assessment of heavy metals in soil and water from Bahi district, Tanzania
Source: PLoS One. 2025 Jun 11;20(6):e0325487. doi: 10.1371/journal.pone.0325487 (PMC12157122; doi:10.1371/journal.pone.0325487)
Supplement: S1 Fig — (PDF) [file pone.0325487.s002.pdf]

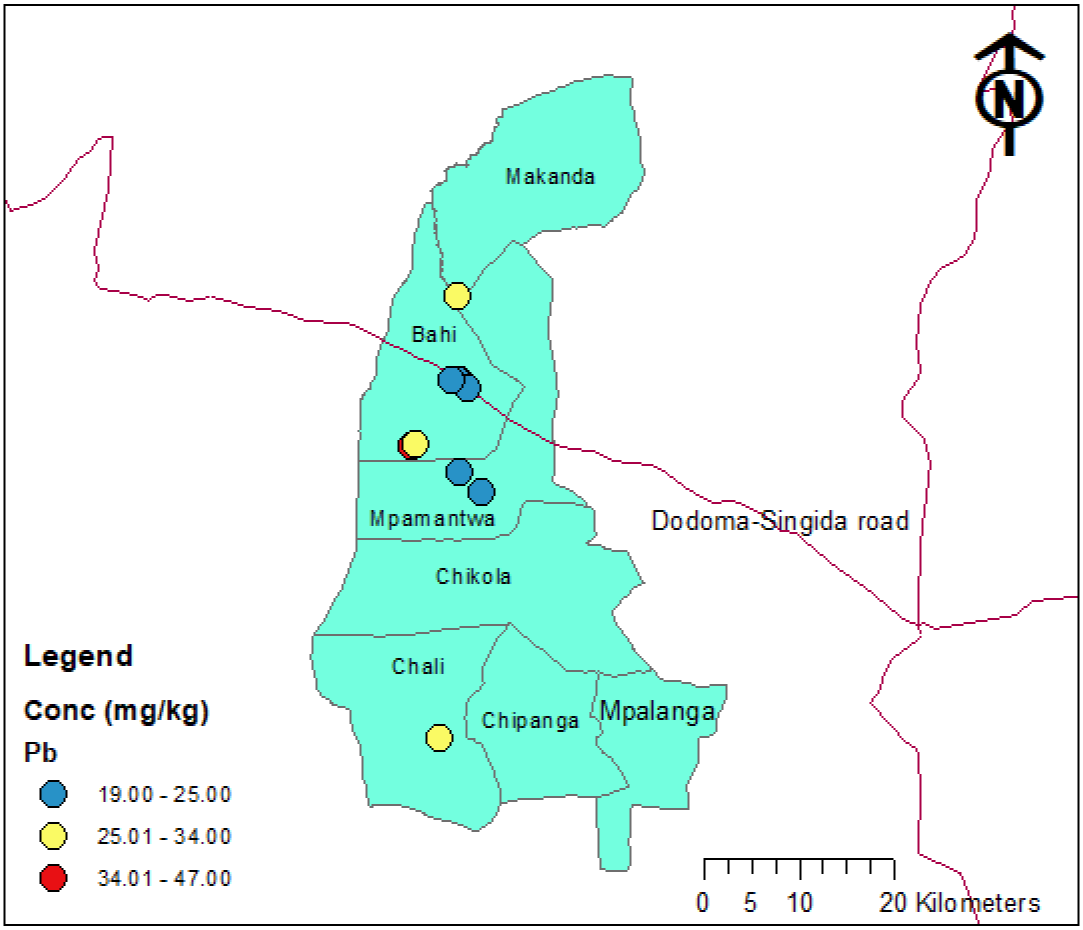

S1A Fig. Map showing Pd concentrations range (mg/kg) for different sampling points.

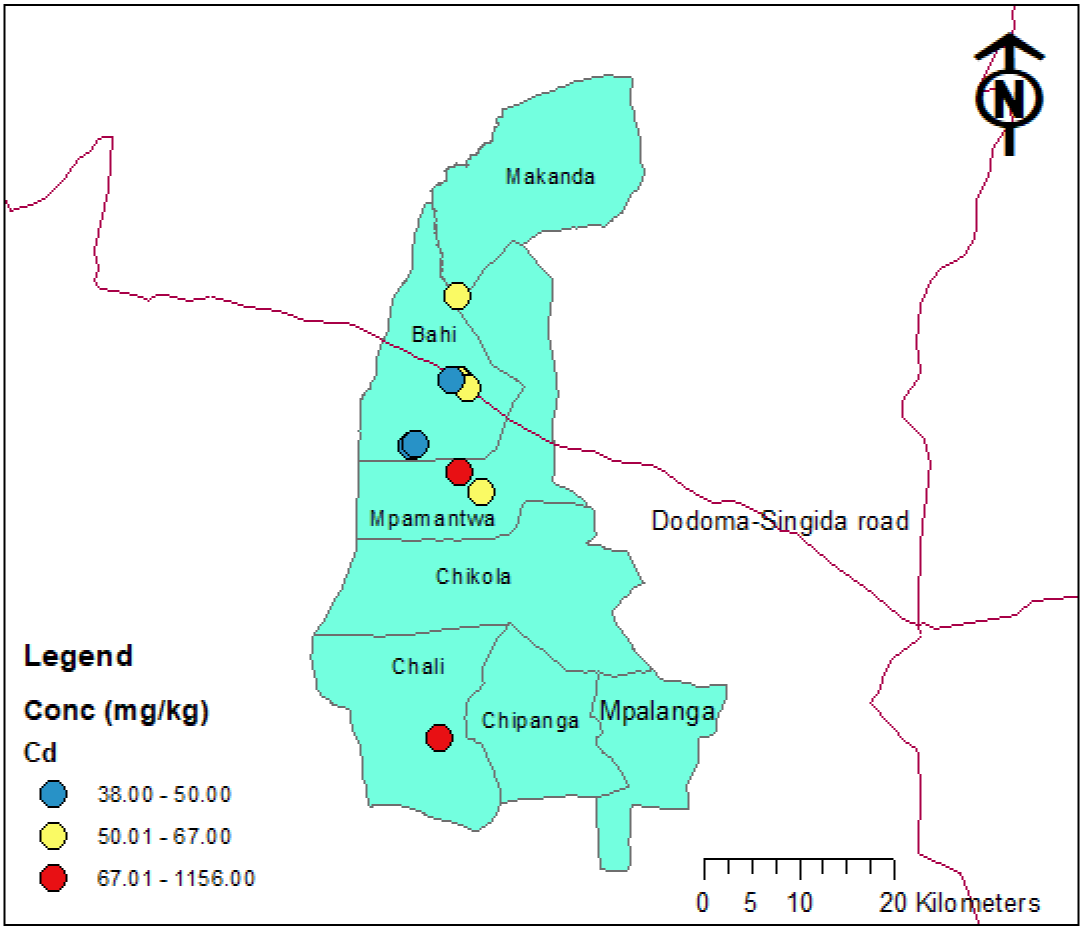

S1B Fig. Map showing Cd concentrations range (mg/kg) for different sampling points.

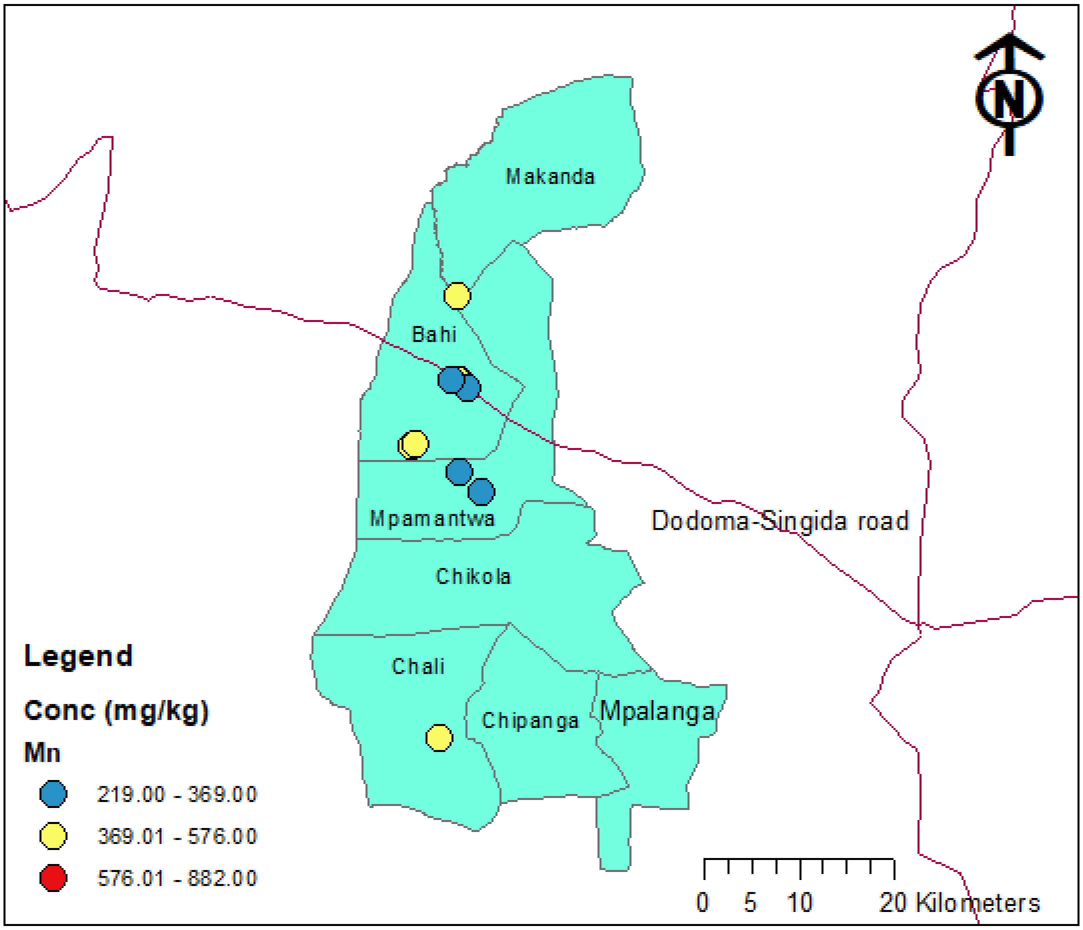

S1C Fig. Map showing Mn concentrations range (mg/kg) for different sampling points.

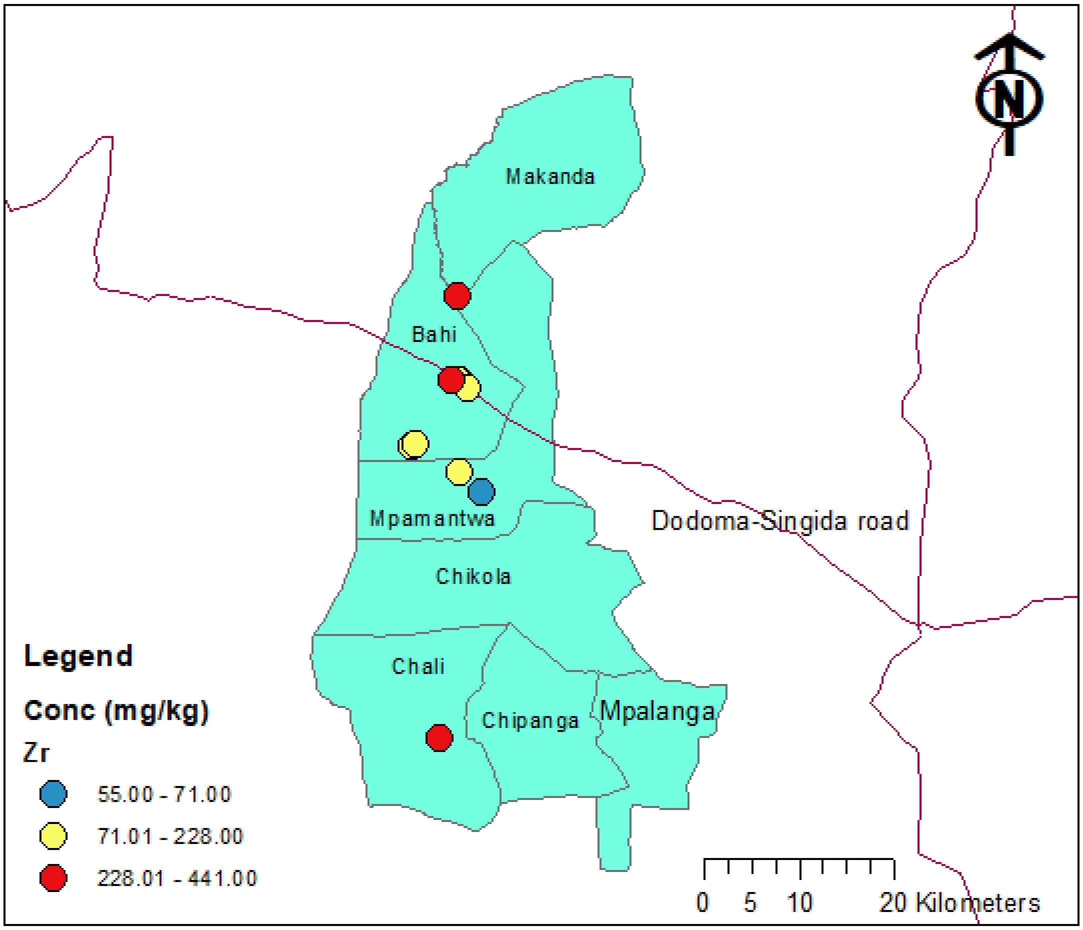

S1D Fig. Map showing Zr concentrations range (mg/kg) for different sampling points.

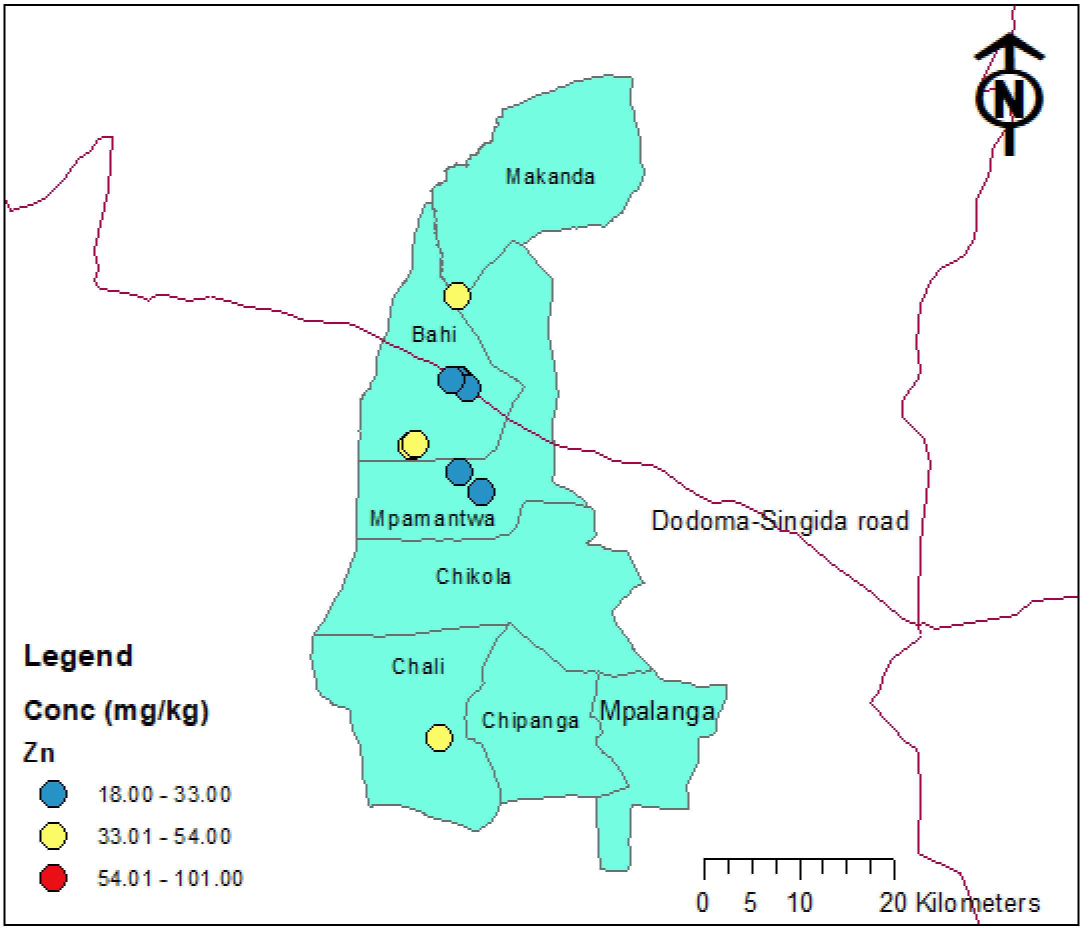

S1E Fig. Map showing Sr concentrations range (mg/kg) for different sampling points.

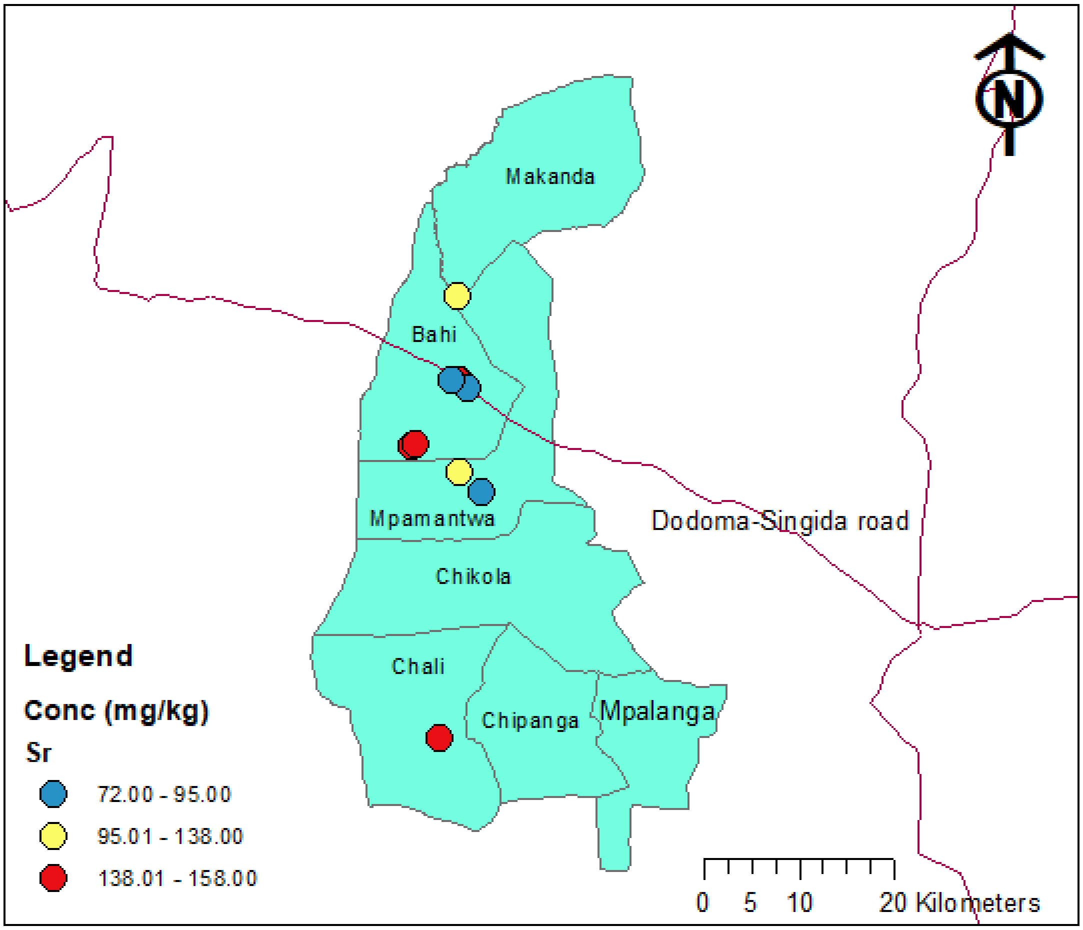

S1F Fig. Map showing Sr concentrations range (mg/kg) for different sampling points.

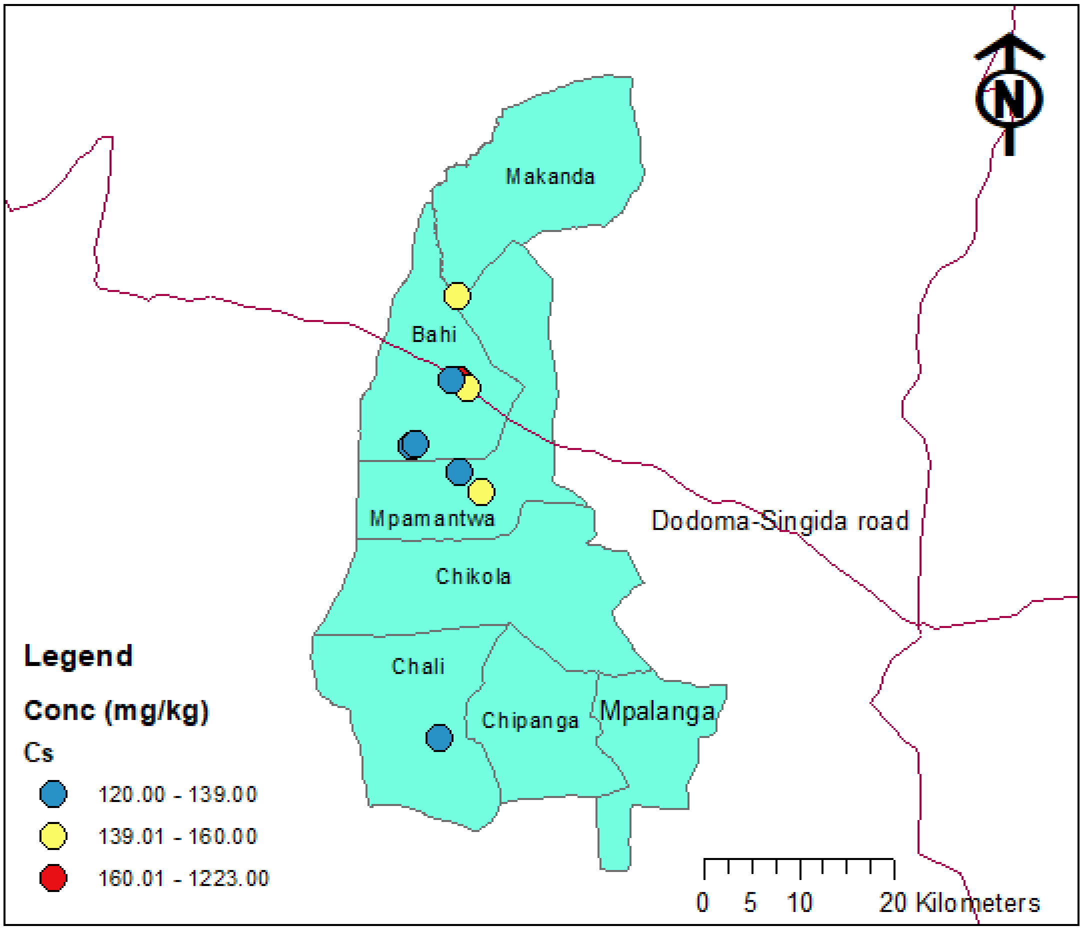

S1G Fig. Map showing Cs concentrations range (mg/kg) for different sampling points.

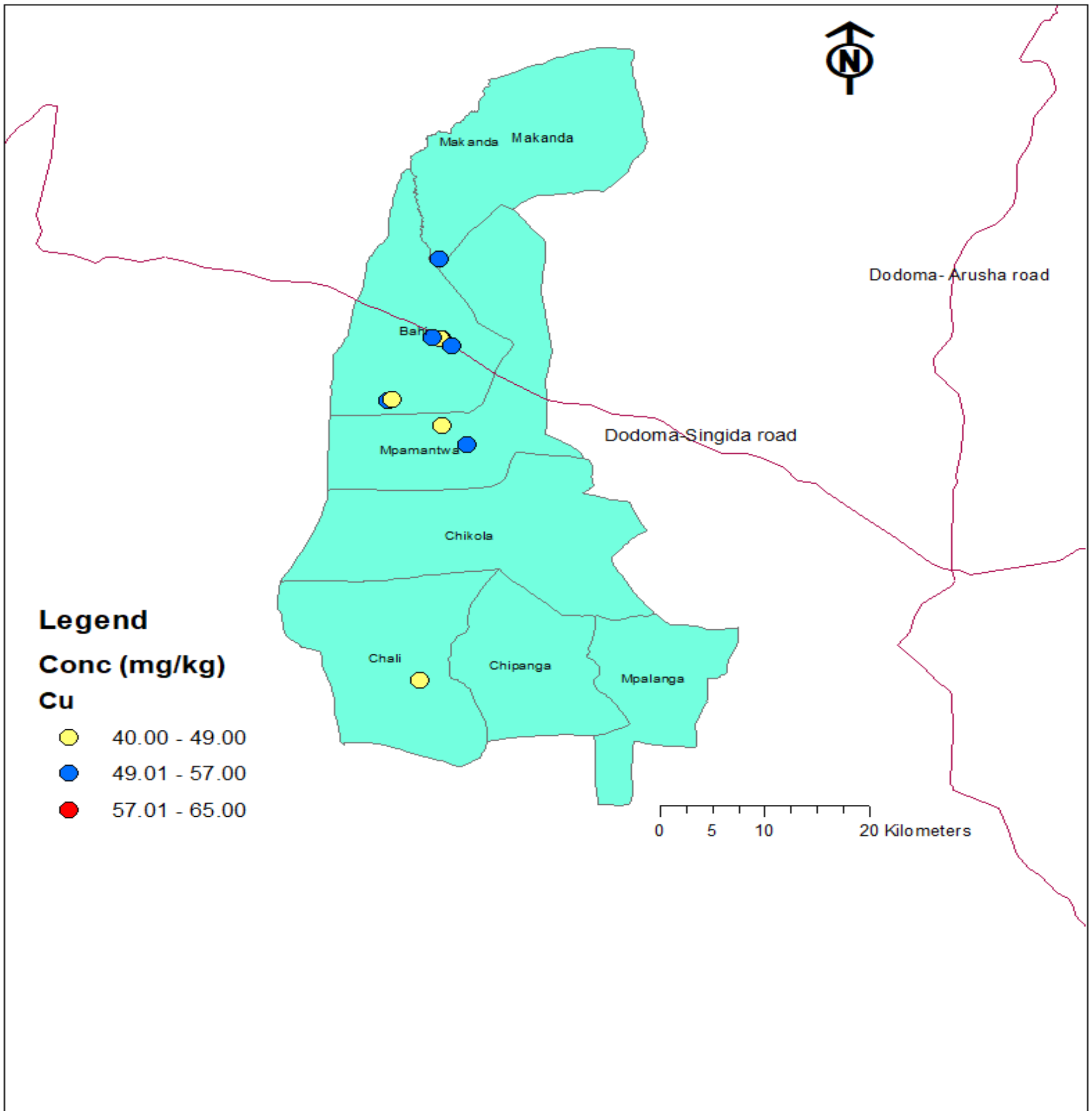

S1H Fig. Map showing Cu concentrations range (mg/kg) for different sampling points.
