# Supplementary material for: Assessment of heavy metals in soil and water from Bahi district, Tanzania
Source: PLoS One. 2025 Jun 11;20(6):e0325487. doi: 10.1371/journal.pone.0325487 (PMC12157122; doi:10.1371/journal.pone.0325487)
Supplement: Attachment — Submitted filename: Response to reviewers comments.docx [file pone.0325487.s004.docx]

PONE-D-24-37705 : Assessment of heavy metals in soil and water from Bahi district, Tanzania

RESPONSE TO JOURNAL REQUIREMENTS:

Item #	Concern/response/action

Comment 1. Please ensure that your manuscript meets PLOS ONE's style requirements, including those for file naming. The PLOS ONE style templates can be found at

https://journals.plos.org/plosone/s/file?id=wjVg/PLOSOne_formatting_sample_main_body.pdf and

https://journals.plos.org/plosone/s/file?id=ba62/PLOSOne_formatting_sample_title_authors_affiliations.pdf

**Action taken. PLOS ONE's style requirements observed**

Comment 2: In your Methods section, please provide additional information regarding the permits you obtained for the work. Please ensure you have included the full name of the authority that approved the field site access and, if no permits were required, a brief statement explaining why.

**Response: Included**

Comment 3: We note that the grant information you provided in the ‘Funding Information’ and ‘Financial Disclosure’ sections do not match.

**Response : Not clear, about the miss-match**

Comment

When you resubmit, please ensure that you provide the correct grant numbers for the awards you received for your study in the ‘Funding Information’ section.

**Response: Not applicable**

Comment 4. We note that your Data Availability Statement is currently as follows: All relevant data are within the manuscript and its Supporting Information files.

**Response: All relevant data included in the manuscript or in form of supporting information**

Comment

Please confirm at this time whether or not your submission contains all raw data required to replicate the results of your study. Authors must share the “minimal data set” for their submission. PLOS defines the minimal data set to consist of the data required to replicate all study findings reported in the article, as well as related metadata and methods (https://journals.plos.org/plosone/s/data-availability#loc-minimal-data-set-definition).

For example, authors should submit the following data:

- The values behind the means, standard deviations and other measures reported;

- The values used to build graphs;

- The points extracted from images for analysis.

Authors do not need to submit their entire data set if only a portion of the data was used in the reported study.

**Action taken: Data available on manuscript or in form of supporting information**

Comment

If your submission does not contain these data, please either upload them as Supporting Information files or deposit them to a stable, public repository and provide us with the relevant URLs, DOIs, or accession numbers. For a list of recommended repositories, please see https://journals.plos.org/plosone/s/recommended-repositories.

**Response : Implemented**

Comment

If there are ethical or legal restrictions on sharing a de-identified data set, please explain them in detail (e.g., data contain potentially sensitive information, data are owned by a third-party organization, etc.) and who has imposed them (e.g., an ethics committee). Please also provide contact information for a data access committee, ethics committee, or other institutional body to which data requests may be sent. If data are owned by a third party, please indicate how others may request data access.

**Response : Not applicable**

.

ADDITIONAL EDITOR COMMENTS:

Item #	Concern/Response/Action taken

1. Methodology: Elaborate on sampling specifics, quality control, and statistical methods.

**Response : Detailed included.**

2.Data Visualization: Use graphical aids to make data interpretation more intuitive.

**Response : Several figures were reproduced for clarity and by the use of SPACE**

3. Discussion Expansion: Provide a thorough exploration of health and environmental implications, drawing connections with prior research.

**Response :Detailed explanation of the results was considered. Results for each element were discussed**

4. Language and Structure: Address grammatical and structural issues to meet journal standards, including reducing similarity and enhancing originality.

**Response : To the level best of our comprehension we observed language issues.**

RESPONSES TO REVIEWERS’ COMMENTS

Reviewer #1

The manuscript deals with "Assessment of heavy metals in soil and water from Bahi district, Tanzania" the following comments and suggestions are need to address before its consideration.

Comment 1: The methodology section should provide more detailed descriptions of the sampling process, including the specific locations and conditions under which samples were collected. This will help readers understand the context and reliability of the data

**Response 1:	Sampling Locations: We have specified the five villages (Bahi Makulu, Bahi Sokoni, Matajila, Chang’ombe, and Nagulo) where the soil and water samples were collected. These locations have been selected based on their proximity to Bahi Swamp and their significance in the district’s economic activities such as agriculture and fish-keeping.**

**Sampling Conditions: The manuscript now includes details about the sampling conditions, such as the time of collection, the weather conditions during sampling, and the tools and protocols used to prevent contamination during collection.**

**Coordinates: The exact GPS coordinates of each sampling site have been added to Supporting information to further improve the reproducibility of the study**.

Comment 2: While the manuscript mentions the use of descriptive statistics, ANOVA, and PCA, it would be beneficial to elaborate on the rationale for choosing these specific statistical methods. Additionally, including a brief explanation of how these analyses contribute to understanding the relationships between heavy metals and metal oxides would strengthen the analysis section

**Response 2: Comments implemented by revising the with inclusion of justification and explanation.**

Comment 3: More emphasis on the quality control measures taken during the study, such as the use of blank solutions and recovery studies, would add credibility to the results. This could be integrated into the methodology section. https://link.springer.com/article/10.1007/s12011-024-04234-0,

https://www.sciencedirect.com/science/article/pii/S0278691524003727

**Response 2: Familiarized with the content and context to improve the section**

Comment 4 (1): What specific AAS techniques were utilized?

**Response 4 (1): Specific AAS technique used was elaborated in -**

Comment 4 (2): How do findings compare to previous studies?

**Response 4 (2): Discussed table of comparison included**

Comment 4 (3): What quality control measures were implemented? Referred to below papers and cite them

https://www.sciencedirect.com/science/article/pii/S0946672X24001019, https://www.sciencedirect.com/science/article/pii/S0946672X24000749, https://www.sciencedirect.com/science/article/pii/S0889157523008177

**Response 4 (3): Quality control measure explained.**

Comment 5: The results section could be improved by including visual aids such as graphs or tables that summarize the heavy metal concentrations in both soil and water samples. This would make the data more accessible and easier to interpret for readers

**Response 5: Visual aids improved by redrawing and few added in all case where applicable.**

Comment 6: The discussion should delve deeper into the implications of the findings, particularly regarding the potential health risks associated with heavy metal concentrations. It would be useful to compare the results with similar studies in other regions to provide a broader context

**Response 6: The discussion extended for every metal analyzed and compared with other from the literature.**

Comment 7: The manuscript mentions the presence of radionuclides and heavy metals in the Bahi area. Expanding on how these factors interact with local agricultural practices and their potential impact on the community would provide valuable insights.

**Response 7: To remain focused for the objective of this manuscript radionuclides were not discussed, however, overall consequence in highlighted.**

Comment 8: Including a section that outlines potential future research directions based on the findings would be beneficial. This could involve suggestions for long-term monitoring of heavy metal concentrations or studies on the effectiveness of remediation strategies.

**Response 8: Suggested as part of the recommendations.**

Comment 9: Ensure that the manuscript adheres to PLOS ONE's formatting guidelines, including reference style and figure/table formatting. Additionally, proofreading for grammatical errors and clarity will enhance the overall quality of the manuscript.

**Response 9: Comment addressed**

Reviewer #2

The abstract is longer than the introduction, and the introduction lacks a story to support the research study's clear objective.

Comment 1: The abstract is longer than the introduction, and the introduction lacks a story to support the research study's clear objective.

**Response 1: Harmonized to avoid losing the major focus of the manuscript**.

Comment 2: The introduction should include the specific problem of soil facing the issue of heavy metal pollution.

**Response 2: Elaborated to motive of undertaking the study foe levels of heavy metals was due to geological natural of Bahi district which characterized with the presence of uranium deposit and the co-existing of other toxic element was paramount.**

Comment 3: A detailed overview of the problem, the research gap, the objectives, and the significance of your study should be discussed in the introduction.

**Response 3: revised and exploited the context of the manuscript in the introduction section**

Comment 4 :	Introduce Bahi district characteristics.

**Response 4 : Addressed in introduction and in study area section**

Comment 5:The spelling of anthropogenic is wrong in the introduction like anthroponenic*.

**Response 5: Corrected.**

Comment 6:	Lines 92-104 are copied and need to be rewritten

**Response 6: Considered where necessary.**

Comment 7:	Lines 389-392 are copied and need to be re-written

**Response 7: Considered.**

Comment 8:Captions of the tables and figures need to be appropriate

**Response 8: Revised as appropriate.**

Comment 9: In methodology, XRF used for metals composition report complete

**Response 9: Addressed with addition of technical details.**

Comment 10: Geospatial information of water samples is not well-defined

**Response 10: Geospatial information explained.**

Comment 11: Table 1. Maximum standard permissible heavy metal concentrations in soil or water should be soil and water

**Response 11:Corrected**

Comment 12:Figure Captions are not well placed, and figure resolution is not up to the mark

**Response 12:Addressed and some figures where reproduced.**

Comment 13:Maps missing the proper labelling of sampling points

**Response 13:Names or Wards where sampling was carried out are indicated.**
