# Supplementary material for: Assessment of heavy metals in soil and water from Bahi district, Tanzania
Source: PLoS One. 2025 Jun 11;20(6):e0325487. doi: 10.1371/journal.pone.0325487 (PMC12157122; doi:10.1371/journal.pone.0325487)
Supplement: Attachment — Submitted filename: Response -MAY 2025.docx [file pone.0325487.s005.docx]

**RESPONSES TO REVIEWER’S COMMENTS.**

**Reviewer #1:** In my opinion, this new manuscript represents the authors' excellent work. The latest version of the manuscript has improved its quality.

**Response:**

We appreciate for the reviewer’s time and comment for the work done.

**Reviewer #2:** Manuscript lacks the strength in research hypothesis and objective. Research work is just based on very simple approach which do not reflect any potential outcome and reader preference as well as interest.

**Response**

We appreciate the reviewer’s feedback on the research hypothesis and objectives. This study aims to address a gap in the literature on the geochemical distribution of heavy metals in Bahi District, a region with limited research, particularly concerning its uranium deposits. While the approach may seem straightforward, it is informed by the district's distinct geological and geochemical context.

The hypothesis posits that heavy metal concentrations in soils and water are influenced by both natural geological factors and anthropogenic activities, with potential health implications. The objectives were structured to evaluate concentration and distribution, providing baseline data essential for future studies and contributing to environmental chemistry and health research.

Regarding implementation scope, we acknowledge the study does not cover all variables but represents an essential step in understanding Bahi District’s environmental challenges. The findings not only address local concerns but are also relevant to similar semi-arid regions. This research establishes a foundation for future studies on bioaccessibility, source apportionment, and community engagement to mitigate long-term health risks.

We hope that this clarification addresses the reviewer’s concerns and enlightens the significance and potential impact of the study.

**Reviewer #3:** Editor:

***Comment 1: The abstract is rather lengthy. A portion of the abstract ought to be moved into the Materials and Methods and Introduction sections.***

***Response:***

- About **118 words were removed to reduce the length of the abstract while retaining the meaning and context.**
  - The second and third sentence was removed since it also addressed in methodology (in study area section) and in the introduction section, respectively

***Removed-****About 96% of Bahi district is rural area, which the main economic activities are agriculture, fish-keeping and animal husbandry. The aim of this research was to assess the levels and spatial distribution of heavy metals in Bahi district*. –

- The sixth sentence was removed – it is well explained in the methodology part.

***Removed****- “Descriptive statistics; one-way analysis of variance (ANOVA), and Pearson Correlation Coefficient in conjunction with Principal Component Analysis (PCA) were used to determine variations in concentrations from different samples and the association between heavy metals or with metal oxides.”*

- The 10th sentence- shortened by deleting the ending phrase since it does not alter the meaning. Its relevance is captured in the conclusion section

**Removed-** “…*showing non-uniform distribution and indicating various influencing factors.”*

- The third sentence (last but three) was removed. Since the re-phrasal of the last two serve the purpose as concluding phrases.

**Removed-** *“These findings provide valuable insights into heavy metals distribution in the soils and waters sampled from Bahi localities, aiding in understanding environmental and health implications.”*

***Comment 2: The figures resolution of the article is very low especially fig. 3 to fig. 10. I couldn't see what is written on these figures.***

***Response:***

- We decided to remove figs 4 to 10 and shift them to supporting documents as S6-S12 Figs. They can serve the purpose in case the reader would like to see the spatial distribution details. S1 Fig included in the list hence it is now S13 Fig in the list
- Hence the re-assignment of figure number was done Figs 11 – 16 are now identified as Figs 4 – 9, respectively.

***Comment 3:* Language and Style: The writing is mostly clear, but there are areas where grammatical improvements could enhance readability. A thorough proofreading is recommended.**

**Response:**

- The proofreading has been undertaken to clear grammatical issues and typographic errors. This can be follow-up in the track-changes document.

***Comment 4: The conclusion is poor. It should be rewritten according to the significance of the present outcomes in the context. The authors should mention how to achieve the research question mentioned at the end of the introduction. Conclusions must be deeper.***

***Response:***

*The* conclusion *has been re-written to reflect the following;*

- *Significance of outcomes*: Highlighted how the findings address knowledge gaps on heavy metal distribution in Bahi District, emphasizing health risks linked to metal concentrations.
- *Research question:* Explicitly connected findings to the research question, focusing on metal concentrations and distribution
- *Connection to health policies:* Emphasized the integration of geochemical data with public health policy, offering actionable recommendations for mitigation and monitoring.
- *How deeper is the conclusion?* – For now, it highlighted the persistence of cadmium, toxicological concerns of tellurium, and manganese behavior, providing a deeper understanding of the findings.
